# Complete nucleotide sequence of the 16S rRNA from *Lactobacillus paracasei* HS-05 isolated from women’s hands

**DOI:** 10.1186/s13568-015-0158-8

**Published:** 2015-12-10

**Authors:** Woon Yong Choi, Hyeon Yong Lee

**Affiliations:** Department of Biomedical Materials Engineering, Kangwon National University, Chuncheon, 200-701 Korea; Department of Food Science and Engineering, Seowon University, Cheongju, 361-742 Korea

**Keywords:** *Lactobacillus paracasei* HS-05, Mitochondrial genome, Women’s hand surface

## Abstract

We determined the complete nucleotide sequence of the 16S rRNA from a new bacterium collected from the surfaces of women’s hands. We also compared the presence of various bacteria based on the subjects’ sex and age. The number of colonies isolated from the hand surface was larger for women than men, and the largest number of isolates was confirmed to be present for the women in their 30 s and men in their 40 s (147 and 34 isolates, respectively). The morphology of an isolated bacterial strain was found to be rod type, and the bacterium was identified as *Lactobacillaceae* species based on the GenBank database, through a phylogenetic analysis using the 16S rRNA sequence. Based on the results of a homology search, the isolated strain was 99 % identical to *Lactobacillus paracasei*, so it was designated *Lactobacillus paracasei* HS-05 and was registered in the Korea Culture Center of Microorganisms (KCCM) database as [KCCM11349P].

## Introduction

In addition to their use in fermenting milk products or natural substances, lactic acid bacteria have been reported to be able to perform other special activities, such as producing antibiotics (Lim et al. 2008). In addition, lactic acid bacteria are introduced into the intestines to improve the properties of the intestinal microflora, where they play beneficial roles in host animals, such as stabilization of the intestinal microflora, disease prevention by suppressing the settlement of harmful bacteria, immune activation, anticancer activity, and lowering the LDL cholesterol level (Cotter et al. 2005; Jack et al. 1995; Kojic et al. 1991; Maeng et al. 1997; Marie et al. 1996). Among the substances produced by lactic acid bacteria, bacteriocin is a natural antibiotic protein or protein-based substance and is known to have effective germicidal activity against pathogenic microorganisms (Holo et al. 1991; Petersen et al. 2006; Schillinger and Lucke 1989; Todorov and Dicks 2005).

In addition to their applications in fermentation, lactic acid bacteria are being increasingly utilized in the cosmetics industry. Lactic acid bacteria fragments have shown antioxidant effects and whitening effects, and they increase the activity of cosmetic agents (Choi et al. 2013). However, to date, the lactic acid bacteria that have been used for cosmetics were those that were already in use in food fermentation, such as *Lactobacillus rhamnosus*, *Lactobacillus paracasei*, and *Lactobacillus casei* (Frederic and Vuyst 2004). Therefore, lactic acid bacteria that better fit the characteristics needed for the cosmetics industry should be identified to provide more effective activities.

The human skin contains numerous microorganisms, with the *Propionibacterium, Streptococcaceae*, *Staphylococcaceae,* and *Lactobacillaceae* known to exist in the largest numbers. Among these, *Propionibacterium* and *Streptococcaceae* have been reported to be predominant, with the *Lactobacillaceae* comprising a smaller population (Fierera et al. 2008). However, among the various human body parts, lactic acid bacteria (especially *Lactobacillaceae*) are found in large numbers on the hands, and it has previously been shown that more lactic acid bacteria are present on women’s hands than on men’s hands (Fierera et al. 2008; Costello et al. 2009; Dong et al. 2011). If new human skin-derived lactic acid bacteria can be isolated and identified, they can be utilized in the development of natural food preservatives in the current probiotics market and as alternative medicines (to provide antibiotics), and moreover, these bacteria may be more appropriate for use in the cosmetics industry. Therefore, in the present study, a new human skin-derived *Lactococcus paracasei* strain, HS-05, was isolated, and its morphological characteristics were investigated. In addition, 16S rRNA sequencing was conducted to identify the microorganisms isolated from human hands.

## Materials and methods

### The isolation of a new bacterial strain from human hands

To isolate lactic acid bacteria from human skin, approximately 40 men and women in their teens to their 40 s were asked to participate in an experiment 7 days prior to the experiment being performed. The experiment was conducted on an unspecified day so the participants would not wash or treat their hands differently before the experiment (Fierera et al. 2008). The right palm of each participant was pressed on MRS Agar (288210, Difco, USA), which was made in advance to inoculate the culture medium with initial microorganisms, and the inoculated initial samples were cultivated for 48 h in 37 °C incubators using anaerobic gas packs (Gas Pak, BBL, USA). After the culture, the obtained microorganisms were dispensed by streaking with platinum loops on MRS Agar and were cultured under anaerobic conditions for 48 h in 37 °C incubators to obtain individual strains. Using the isolated lactic acid bacteria, the phylogenetic tree of the 16S rRNA sequences was analyzed (Baek et al. 2010). For more detail characterization of the bacterium, the changes of cell growth and pH in the medium were also measured according to the culture temperature since the temperature of the hands was mostly affected under conventional environments. First, the cultures of the bacterium were incubated at 25, 30, 37, 40 and 45 °C for 24 h. At the end of each incubation time, the counts of surviving cells were determined by plating on MRS agar. In addition, the pH (initial pH of the medium was 6.5) was also measured for 24 h cultivation by a pH meter (Sentron Titan pH meter) (Tomas et al. 2003).

### Analysis of the phylogenetic tree of the 16S rRNA sequences

To compare the phylogenetic trees of the isolated bacteria, a 16S rRNA analysis was conducted using the iQ5 real-time PCR detection system (Bio-Rad Laboratories). The phylogenetic tree was generated through a molecular phylogenic analysis based on the 16S rRNA gene base sequences of the isolated strains. The primers used to amplify the16S rRNA genes were the 27F (5′-AGAGTTTGATCMTGGCTCAG-3′) primer and 1492R (5′-TACGGYTACCTTGTTACGACTT-3′) primer synthesized based on the conserved sequence of the *E. coli* 16S rRNA gene. The RT-PCR was conducted by performing 33 cycles of denaturation (94 °C, 30 s), annealing (60 °C, 30 s), and elongation (72 °C, 45 s), followed by incubation for 15 min at 72 °C (Baek et al. 2010). After determining the 16S rRNA gene nucleotide sequences, each base sequence was compared with the base sequences of similar strains in the GenBank database to determine the phylogenetic locations of the strains (Baek et al. 2010).

### Scanning electron microscopy (SEM)

To photograph the newly isolated bacterial strain, a low-vacuum scanning electron microscope (SEM) (XL 30, Philips, The Netherlands) was used at 400× magnification to observe the morphology of the bacteria. Sample slices were immersed in an 8 % paraformaldehyde and 2.5 % glutaraldehyde solution made using 0.05 M sodium cacodylate buffer (pH 7.2) and were fixed four 2 h at 4 °C. The sample slices were washed three times for 2 min each time using 0.05 M sodium cacodylate buffer (pH 7.2) and were then fixed by treatment in a 1 % osmium tetroxide solution for 2 h at 4 °C, followed by two washes with tertiary distilled water at room temperature. The fixed samples were dehydrated in 30, 50, 70, 80, and 90 % ethanol for 10 min each and in 100 % ethanol three times for 10 min each time. After the dehydration, the samples were mounted on metal stubs by two transitions for 15 min using 100 % propylene oxide and were then coated with gold using a sputter coater (Agar Scientific Ltd. SC502, USA). The samples were observed using a scanning electron microscope (Philips XL30E, USA) (Williams and Davies 1967).

## Results

### Isolation and characteristics of a new bacterial strain

**Fig. 1:**
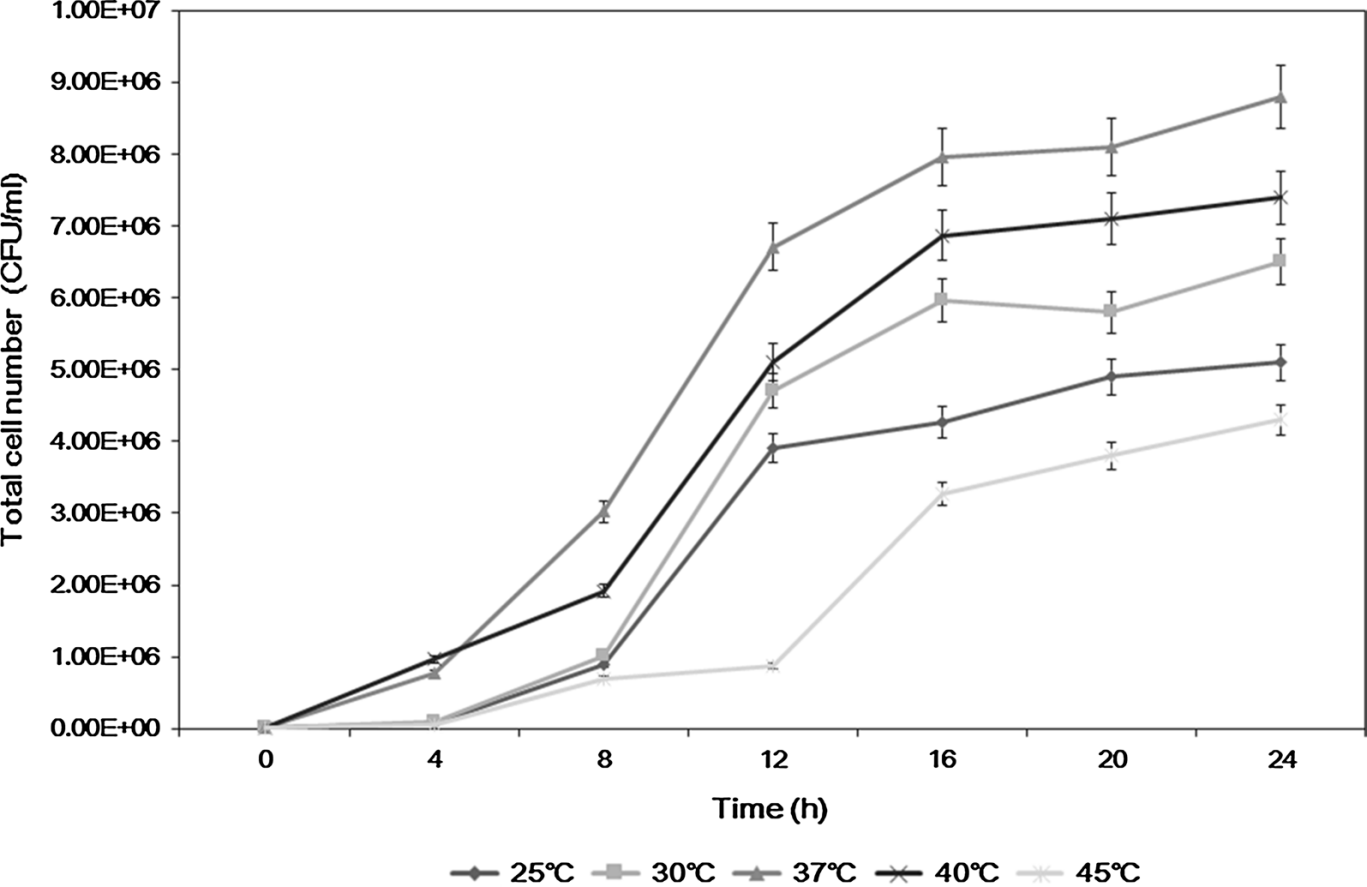
The growth of *Lactobacillus paracasei* HS-05 in MRS broth at different culture temperatures

**Fig. 2:**
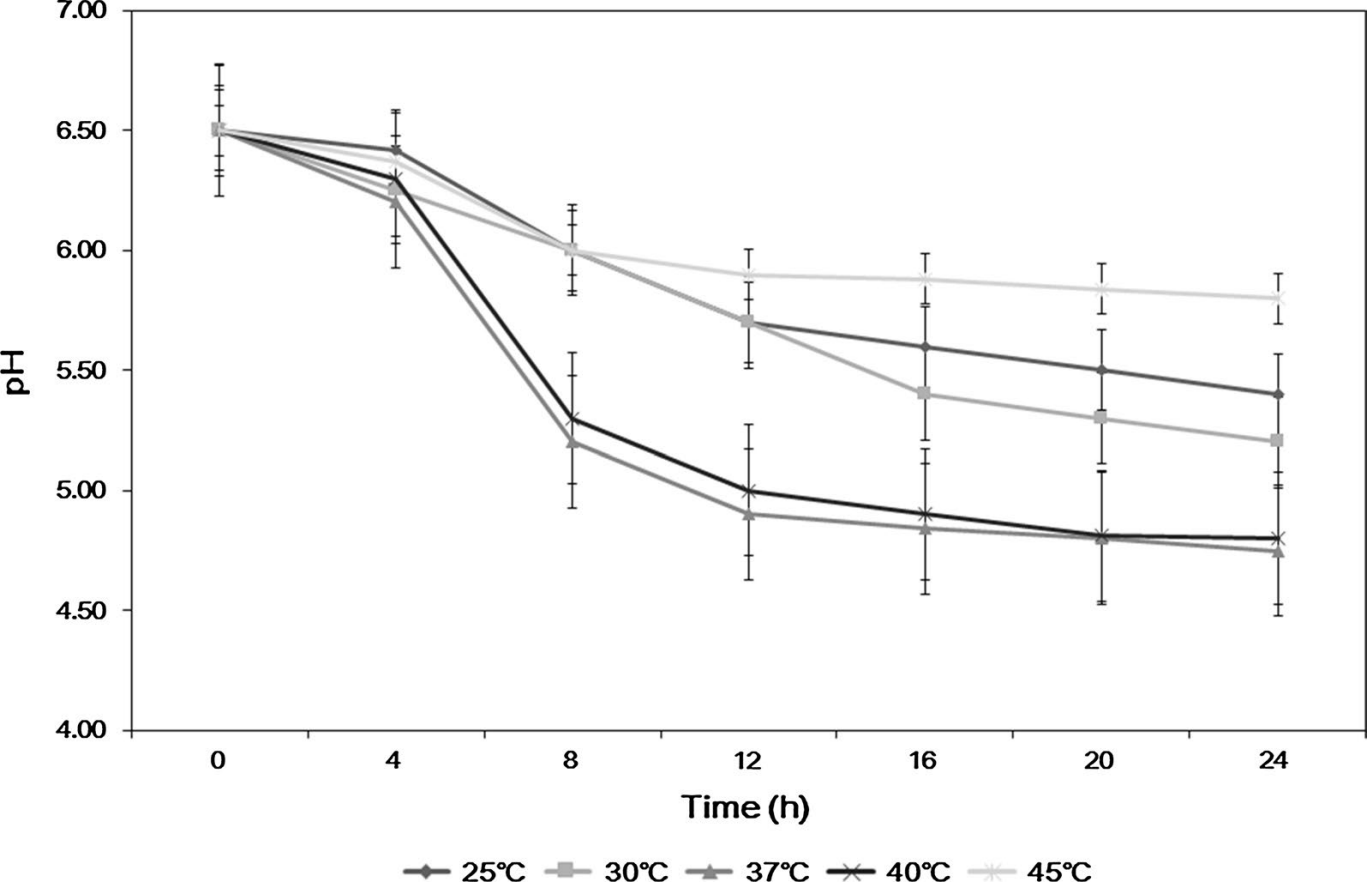
pH changes of the culture broth of *Lactobacillus paracasei* HS-05 according to different culture temperatures

**Fig. 3:**
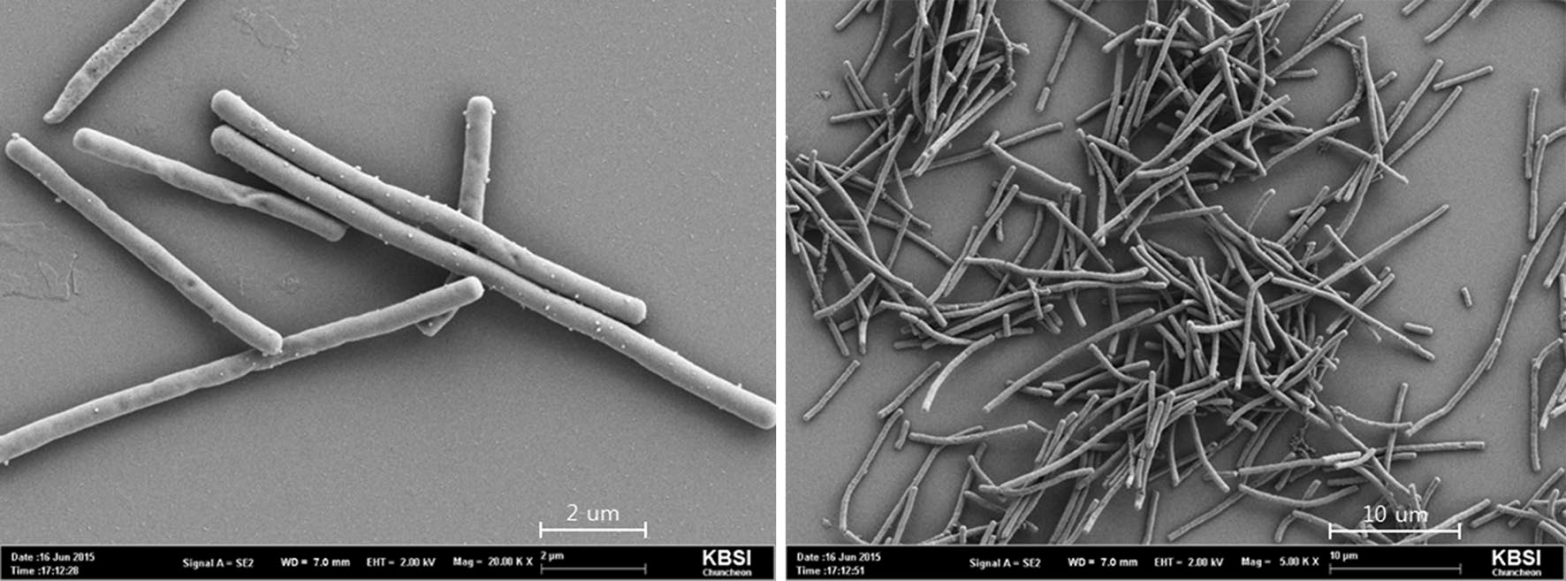
A field emission scanning electron microscope (FE-SEM) image of *Lactobacillus paracasei* HS-05

Lactic acid bacteria were isolated by pressing the unwashed hands of men and women in their 10–40 s on MRS (de Man, Rogosa and Sharpe) agar culture medium. The numbers of each age of men and women (ages of 10, 20, 30 and 40’s) in the experiments were 25 and the mean averages of the colonies in the agar plates from each age were estimated after counting the colonies. The proband of all the examinees was varied such as secretaries, students, hard workers, and drivers, etc. and the terms of their jobs were also varied from less than 1–20 years, which should affect the bacterial profiles of their hands. The numbers of colonies obtained from the men and women were compared (not shown data). Based on the results, the average number of colonies of men in their teens was 56, and that of women in their teens was 51; further, that of men in their 20 s was 47, and that of women in their 20 s was 77. Although the number of colonies obtained from men in their 30 s dramatically decreased down to ca. 23, the number obtained from women in their 30 s was approximately 147. In addition, the number of colonies from men in their 40 s was ca. 34, while the number from women in the same ages was decreased down to only 58. Based on these results, we concluded that the number of colonies isolated from the hands of women tended to be higher than that isolated from men, and when the numbers of colonies were compared by age group, it was found that women in their 30 s had the largest number of colonies. A total of four subcultures from the colonies obtained from women were conducted on MRS Agar culture medium, and the bacteria that formed clear white colonies were selected. For further characterization of the bacterium, as shown in Fig. 1, optimal growth temperature was determined as 37 °C, which is closed to human temperature, by showing that 6.7 × 10^6^ viable CFU/mL were observed after 12 h cultivation at 37 °C while about 7.4–8.8 × 10^6^ CFU/mL of cell density were maintained for 24 h at a temperature of 37–40 °C. In addition, Fig. 2 shows the change of pH in the medium according to the culture temperature, and measurements are shown in Fig. 2 according to the temperature. At 37 °C, most proper pH for growing lactic acid bacteria as pH 5 was maintained by comparing the pH of the medium under other culture temperature (Adamberg et al. 2003), which implied that most vital cells could be observed at the temperature between 37 and 40 °C. These results could also tell that an optimum culture condition for this bacterium was 37 °C and pH 5. Figure 3 shows the SEM analysis for the morphological identification of the selected bacteria, and the new strain was identified as a rod-type lactic acid bacteria. Thereafter, the bacteria were amplified using PCR to analyze the 16S rRNA to conclusively identify the strain.

### Analysis of the phylogenetic tree of the 16S rRNA sequence of the new strain

**Fig. 4:**
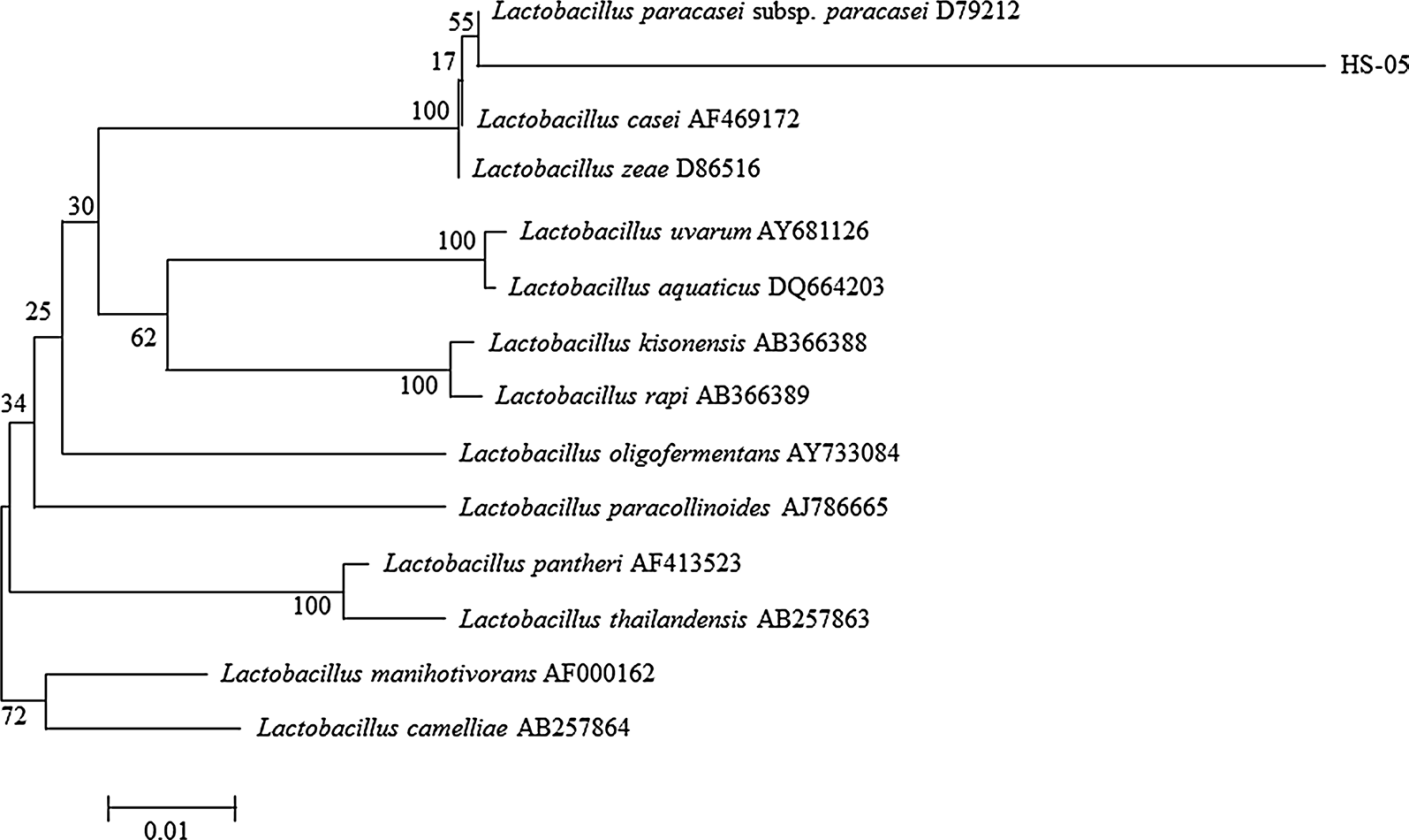
The results of the phylogenetic tree analysis of *Lactobacillus paracasei* HS-05 based on 16S rRNA gene sequences

To identify the isolated strain, the base sequence of the 16S rRNA gene was analyzed, and the results are shown in Table 1. The analyzed base sequence was compared with the base sequences of similar strains registered in the GenBank database to examine the correlations between genes. Based on the results, the isolated strain had 55 % homology with *Lactobacillus paracasei* subsp. *Paracasei* D79212. In addition, to determine the strain’s molecular phylogenic relationship with the existing *Lactobacillus* group based on the structure of the 16S rRNA gene, a phylogenetic tree was prepared, and the results are shown in Fig. 3. The molecular phylogenic analysis also showed that the isolated strain fell under the phylogenetic group that included *Lactobacillus paracasei*. Therefore, the lactic acid bacteria that was isolated from women’s hands was named *Lactobacillus paracasei* HS-05 (Fig. 4).

**Table 1:** The complete nucleotide sequence of the 16S rRNA from *Lactobacillus paracasei* HS-05

HS-05	
AAGATTCTGTCAACAACGGTATCCATATGAGTTTGATCATGGCTCAGGAAGTCGTAACAAG GTGTCCATAGAGTTTGATCATGGCTCACGATATCTTAACACGGTGTCTCTATAGTTTGTGCT TGGCTCACAAAGACTCAACAACGGGTCGGTACATGTTTGAAATATGGGTAAGCTATCGCTT TTGGATGGACCCACGGCGTATTAGCTAGTTGGTGAGGTAATGGCTCACCAAGGCGATGATAC GTAGCCGAACTGAGAGGTTGATCGGCCACATTGGGACTGAGACACGGCCCAAACTCCTACG GGAGGCAGCAGTAGGGAATCTTCCACAATGGACGCAAGTCTGATGGAGCAACGCCGCGTGA GTGAAGAAGGCTTTCGGGTCGTAAAACTCTGTTGTTGGAGAAGAATGGTCGGCAGAGTAACT GTTGTCGGCGTGACGGTATCCAACCAGAAAGCCACGGCTAACTACGTGCCAGCAGCCGCGG TAATACGTAGGTGGCAAGCGTTATCCGGATTTATTGGGCGTAAAGCGAGCGCAGGCGCTTTT TTAAGTCTGATGTGAAAGCCCTCGGCTTAACCGAGGAAGCGCATCGGAAACTGGGAAACTT GAGTGCAGAAGAGGACAGTGGAACTCCATGTGTAGCGGTGAAATGCGTAGATATATGGAAG AACACCAGTGGCGAAGGCGGCTGTCTGGTCTGTAACTGACGCTGAGGCTCGAAAGCATGGGT AGCGAACAGGATTAGATACCCTGGTAGTCCATGCCGTAAACGATGAATGCTAGGTGTTGGA GGGTTTCCGCCCTTCAGTGCCGCAGCTAACGCATTAAGCATTCCGCCTGGGGAGTACGACCG CAAGGTTGAAACTCAAAGGAATTGACGGGGGCCCGCACAAGCGGTGGAGCATGTGGTTTAA TTCGAAGCAACGCGAAGAACCTTACCAGGTCTTGACATCTTTTGATCACCTGAGAGATCAG GTTTCCCCTTCGGGGGCAAAATGACAGGTGGTGCATGGTTGTCGTCAGCTCGTGTCGTGAGA TGTTGGGTTAAGTCCCGCAACGAGCGCAACCCTTATGACTAGTTGCCAGCATTTAGTTGGGC ACTCTAGTAAGACTGCCGGTGACAAACCGGAGGAAGGTGGGGATGACGTCAAATCATCATG CCCCTTATGACCTGGGCTACACACGTGCTACAATGGATGGTACAACGAGTTGCGAGACCGC GAGGTCAAGCTAATCTATTAAAGCCAATATCAGTTCAGGAGAGTAGGCTGCAACTCGCCTA CGTGAAATCGGAATCTCCAGAGAGTGCGAATCAGCATGACGAAGTGATAACAATGTCTCCC TAGAGTCAGATCACGGCTCAGCAAGTCGTAACAAGGTATCCATAGAGTTTGATCGAGGTCT CCAGCTAAAGTTCCG

## Discussion

From this work, a human hand-derived lactic acid bacterium was successfully isolated. Although *Proprionibacteria* and *Streptococcaceae* are known to be present in large numbers on human skin, the *Lactobacillaceae* were found to be distributed in large numbers on the human hands (Fierera et al. 2008). In addition, more colonies were cultured from women’s hands than from men’s hands, and this is considered to be attributable to the fact that unlike the other types of common skin bacteria, the number of *Lactobacillaceae* are more variable and are not evenly distributed between men and women (Fierera et al. 2008). In particular, large numbers of colonies were observed for the hands of women in their 30 s, which was considered to be due to the fact that their frequency of contact with cosmetics and/or foods is high (Costello et al. 2009; Dong et al. 2011). From the result of this work, *Lactobacillus paracasei* HS-05 was not found on men’s hands but was found on women’s hands. Moreover, it was also first confirmed that the bacterium profile and existence of *Lactobacillus paracasei* HS-05 were not much affected by the personal histories and their job careers by having consistently high numbers of the colonies in most groups of women hands, not any kinds of men’s hands. It is very interesting that the dominant existence of specific lactic acid bacterium was observed on the women and specially for young age women, regardless of their jobs, which could be further utilized for isolating other specific purpose bacterium from human body.

*Lactobacillus paracasei* HS-05 bacteria could be identified through a 16S rRNA analysis, as shown in Table 1, and no strain that has the same specific base sequence has been reported yet (Janda and Abbott 2007). Therefore, based on the 16S rRNA analysis and the molecular phylogenic analysis, the isolated strain was considered to fall under the phylogenic group that includes *Lactobacillus*, and had 99 % homology with *Lactobacillus paracasei* subsp. *Paracasei* D79212. This is the first report of the existence of this strain on the hand surface of women, and this bacterium was thus named *Lactobacillus paracasei* HS-05 and has been registered in the Korea Culture Center of Microorganisms (KCCM) as [KCCM11349P].
